# Supplementary material for: Protocol for a scoping review of time to treatment in adults with newly diagnosed multiple myeloma
Source: PLoS One. 2025 Aug 25;20(8):e0330907. doi: 10.1371/journal.pone.0330907 (PMC12377623; doi:10.1371/journal.pone.0330907)
Supplement: Attachment — Submitted filename: Response Letter.docx [file pone.0330907.s002.docx]

July 11, 2025

Carmelo Caldarella, Ph.D., M.D.
Academic Editor, PLOS ONE

RE: Manuscript ID PONE-D-25-22988

Greeting Dr. Caldarella,

Thank you to the editors and the reviewers for the thoughtful feedback on our scoping review titled *‘Protocol for a scoping review of time to treatment in adults with newly diagnosed multiple myeloma’.* Thank you also, for allowing us the opportunity to improve our manuscript and resubmit. Below is a summary of reviewers’ comments and our responses. Please note that reviewers 1 and 4 raised no concerns and are therefore not represented in our summary. We feel that we have responded appropriately to reviewers’ concerns and that our manuscript has improved as a result. Thank you for your consideration.

On behalf of the authors,

Matthew R. LeBlanc PhD, BSN

Assistant Professor

School of Nursing

University of North Carolina at Chapel Hill

| **Reviewer 2 Comments** | |
| --- | --- |
| 1. Given the well-documented differences in outcomes between transplant-eligible and non-eligible patients, I recommend stratifying the study population into at least two groups to evaluate overall survival (OS) and progression-free survival (PFS) | Thank you for this wonderful suggestion. We have added language to our analysis section that indicates that we will stratify results by transplant eligibility as the data allows. (page 6) |
| 2. If feasible, the data should also be stratified by treatment regimen—for example: VTD, VRD, RD, VD, VMP vs daratumumab-based or other monoclonal antibody-based regimens vs others. | We have added language to our analysis section indicating that we will stratify results by induction regimen as data allows. |
| **Reviewer 3 Comments** | |
| 1. Clarify how differences in definitions of TTI will be managed during analysis. Will you categorize or synthesize them descriptively? | Definitions will be explored descriptively. Results will not be combined as in a meta-analysis. This clarification is on page 6 (Data Analysis and Presentation). |
| 2. Discuss how language restrictions will be handled if English translations are not available. | We do not have the capacity to translate sources and therefore these sources without English translations will be excluded. (See page 4 for added language). |
| 3. Expand on how you plan to address publication bias and the potential variability in study quality | We do not plan on assessing for bias or study quality. Not assessing for bias/quality is common in scoping reviews and would be quite tricky in our case given the mix of study types we plan on including. |
| 4. Include a brief discussion of the anticipated contribution of this scoping review to clinical practice guidelines or research agendas. | Thank you for the invitation to clarify the impact we intent this work to have. We have added language to the Background (page 3) and to the Discussion (page 6) |
| 5. Provide further rationale for the year 2000 as the search cut-off, beyond the general availability of newer treatments | Thank you for the encouragement to clarify our choice here. We have added language to page 4 under ‘Search Strategy’ highlighting how changes in the treatment landscape have changed the cost/benefit of early treatment greatly increasing the rationale and appetite for earlier treatment. |
| 6. Edit the abstract for clarity—avoid phrases like 'surgery' for describing transplantation. | We appreciate the careful reading of our manuscript. In this case it appears there was some mistake. We do not use the word surgery in the abstract or body of our paper. |
| 7. In the methods, consider explaining whether reviewers will be blinded during abstract screening. | We have added language (page 5), clarifying that reviewers will not be blinded during the screening process |
| 8. There is a duplicated reference (LeBlanc 2022, R6-8) that should be consolidated. | Thank you for your close reading of our manuscript. This duplication has been removed. |
| 9. Include the final version of your PRISMA-ScR checklist as an appendix. | We have included a our PRISMA-P checklist as an appendix. (Appendix I) |
| 10. Table 1 could benefit from improved formatting for clarity in separating concepts and terms. | Our table has been updated to clarify the concepts presented. (page 7) |
